# A Systematic Review of Metacognitive Beliefs in Chronic Medical Conditions

**DOI:** 10.3389/fpsyg.2019.02875

**Published:** 2020-01-10

**Authors:** Vittorio Lenzo, Alberto Sardella, Gabriella Martino, Maria C. Quattropani

**Affiliations:** ^1^Department of Human, Social and Health Sciences, University of Cassino and South Latium, Cassino, Italy; ^2^Department of Clinical and Experimental Medicine, University of Messina, Messina, Italy

**Keywords:** metacognition, MCQ-30, cognitive attentional syndrome (CAS), metacognitive beliefs, chronic medical conditions, anxiety, depression

## Abstract

**Background:** Psychological functioning plays an important role in medical conditions and impacts patients' quality of life. Previously, many studies have highlighted the association of metacognition to both the development and maintenance of emotional disorders. Recently, several researchers pointed out the relevant role of dysfunctional metacognitive beliefs in the context of chronic diseases. Hence, dysfunctional metacognitive beliefs could be directly related to anxiety and depression, regardless of the medical condition's expression. The aim of this systematic review was to summarize the available evidence regarding the association of metacognition with anxiety, depression, and perceived quality of life, in the context of medical conditions, according to Wells' theory.

**Methods:** A systematic review based on electronic bibliographic databases (PsycINFO, PubMed, Scopus, Web of Science, and Web of Knowledge) of scientific literature was carried out. Studies involving patients evaluated in clinical settings were included in the analysis.

**Results:** Our findings indicated that metacognition appears to be related to anxiety, depression, and quality of life in patients with medical chronic conditions. Therefore, dysfunctional metacognitive beliefs might be a relevant factor associated with the process of adapting to illness.

**Conclusions:** The additional evaluation of metacognitive factors in the context of several medical chronic conditions appears valuable. Due to the rising interest in the study of metacognition, suggestions for future research have also been provided.

## Introduction

### Rationale

In the past few decades, the role of metacognition in psychopathology has received increased attention. The term metacognition refers to “the aspect of information processing that monitors, interprets, evaluates, and regulates the contents and processes of its organization” (Wells and Purdon, 1999, p. 103). A growing body of research has highlighted that metacognition is associated with the development and the maintenance of psychological disorders. An important approach in this regard is exemplified by the Self-Regulatory Executive Function (S-REF) model, proposed by Wells and Matthews (1996). The principal feature of this model is that it points out the transdiagnostic process involved in emotional disorders. The main focus is not on the symptoms or the diagnosis, but is instead on the dysfunctional metacognitive beliefs and the emotional self-regulation strategies behind them. In fact, the vulnerability and the prolongation of disorders are associated with a non-specific style of thinking, named cognitive attentional syndrome (CAS) (Wells, 2000a,b). CAS refers to repetitive negative thinking in the process of worrying and ruminating, driven by the positive and negative beliefs about worry, concerns about uncontrollability and danger, and the limitations on executive control. The strategies of pathological worry, rumination, and threat monitor describe positive beliefs, while the beliefs about the danger and the uncontrollability of certain thoughts characterize negative beliefs. Some examples of positive beliefs are: “Worrying helps me cope,” or, “Worrying helps me solve problems.” Examples of negative beliefs are: “My worrying is dangerous for me,” and “My worrying could make me go mad.” Consistent with this metacognitive theory of emotional disorders, a series of self-report instruments for assessing dysfunctional metacognitive beliefs were developed. The Metacognitions Questionnaire (MCQ) and its short version (MCQ-30) measure a range of metacognitive beliefs and processes which are considered relevant to the psychological vulnerability and maintenance of emotional disorders (Cartwright-Hatton and Wells, 1997; Wells and Cartwright-Hatton, 2004; Quattropani et al., 2015).

Earlier studies involving clinical samples have shown that metacognitive beliefs are linked to a wide range of psychopathological conditions, such as anxiety disorder (Wells and Carter, 2001), obsessive-compulsive symptoms (Wells and Papageorgiou, 1998), schizophrenic disorders (Larøi et al., 2004; García-Montes et al., 2006), and anorexia nervosa (Cooper et al., 2007).

Recently, a growing number of studies has investigated the role of metacognition even in non-clinical samples. Main findings pointed out that metacognitive beliefs were significantly associated with either perceived stress or negative emotions (Spada et al., 2008b). In addition, dysfunctional metacognitive beliefs predicted the onset of anxiety and depression symptoms in the context of stressful life events (Yilmaz et al., 2011). Moreover, the negative beliefs factor was the strongest predictor for both anxiety and depression (Spada et al., 2008a).

Researchers are becoming increasingly interested in this field as dysfunctional metacognitive beliefs appear to be common factors across a wide range of psychopathologies (Sun et al., 2017).

Currently there are several studies that have demonstrated that metacognitions are involved in the perpetuation of psychological disorders. Therefore, in recent years, systematic reviews and meta-analyses have been carried out on the basis of the available data. Preliminary results suggested that interventions based on metacognition may be effective in anxiety and depressive disorders treatment (Knowles et al., 2016; Normann and Morina, 2018). The greater prevalence of dysfunctional metacognitive beliefs has recently been associated with clinical psychosis (Sellers et al., 2016, 2018).

In recent years, the role of metacognition was investigated in patients with chronic conditions and their caregivers. Anxiety and depression symptoms are common in a wide range of chronic medical conditions, influencing the patients' quality of life (Marchetti et al., 2017; Catalano et al., 2018; Marchini et al., 2018; Martino et al., 2018, 2019c,d,e; Quattropani et al., 2018a,b; Fantinelli et al., 2019; Lenzo et al., 2019). Dysfunctional metacognitive beliefs could be a relevant factor involved in the development of negative emotions, influencing the adherence to medical treatments. In the light of this perspective, metacognitive beliefs of chronic patients and their caregivers could be a significant factor related to the development of distress. Some researchers have started examining this topic. For example, when patients with multiple sclerosis tend to adopt a dysfunctional metacognitive strategy, metacognition can become a relevant therapeutic tool (Pöttgen et al., 2015). The results of a recent study showed insignificant differences between metacognitive factors of multiple sclerosis patients and healthy subjects. Both conflicting and specific correlations between multiple sclerosis patients and control subjects were found (Quattropani et al., 2018c). In addition, coherent findings were revealed in the first research involving cancer patients (Quattropani et al., 2016). Conditions such as cancer are often characterized by the difficulty in the process of making sense integration of the traumatic event and coping, during the first phase (Martino et al., 2019a,b). The process of “making sense,” as a subjective experience, is an important element in promoting a patient's well-being after a traumatic event such as cancer and its related treatments (Martino and Freda, 2016; De Luca Picione et al., 2017). According to this perspective, metacognitions can play a crucial role in the adaptation process of patients and their quality of life. More generally, a deep understanding of the psychological functioning of patients with chronic medical conditions could be useful in implementing tailored psychological interventions in medical settings (Dicé et al., 2016, 2018, 2019).

Despite the considerable amount of studies conducted in the context of chronic medical conditions, there is still a lack of a rigorous and careful summarization of the evidence.

### Objectives

This systematic review aimed at ascertaining the relevance of dysfunctional metacognitive beliefs among patients with chronic medical conditions and/or their caregivers through the analysis of the studies employing the MCQ and the MCQ-30.

### Research Question

We hypothesized that metacognitive beliefs might interact with the experience of a chronic medical condition and contribute to it worsening, affecting psychological distress in both patients and their caregivers.

## Methods

The authors followed the Preferred Reporting Items for Systematic Reviews and Meta Analyses—PRISMA (Liberati et al., 2009; Moher et al., 2009) guidelines for the drafting of this systematic review.

### Search Strategy and Data Sources

A systematic review of the literature was conducted in two stages. Initially, the studies were identified by searching PubMed, WebOfKnowledge, and Scopus using the following keywords: “metacognition,” “metacognitive beliefs,” “metacognition* questionnaire,” “meta-cognition* questionnaire.” We preliminarily filtered the online search by language (English) and species (Humans). The online search was completed on 30th April 2019.

Eventually, the reference lists of the included studies were examined to identify possible relevant studies missed during the database search.

### Inclusion and Exclusion Criteria

Original papers written in English with an available full text were included in the review. Included articles contained information about the subjects affected by chronic medical conditions and/or the patients' caregivers. Studies which employed the “Metacognitive Questionnaire” (MCQ-65) or its brief variant (MCQ-30) as metacognition measure were also included. This review aimed at generically evaluating metacognition, rather than selectively investigating the specifically related symptoms such as paranoia or delusions. In line with this premise, any other tool aimed at assessing metacognition was excluded.

We also excluded articles primarily involving patients with psychiatric conditions, according to the DSM-5 or ICD-10 (World Health Organization, 1992; American Psychiatric Association, 2013), as well as the studies involving only healthy subjects.

The ones which did not clearly provide data on the subjects' medical conditions or the assessment of metacognition were excluded.

### Eligibility Screening

The eligibility was assessed in a three-step procedure by two different authors (VL, AS): first by the title, then by the abstract, and finally by a full text screening. Conflicts regarding eligibility were resolved by consulting a senior author (MQ).

The review articles were not assessed for eligibility, however they were used as a source for potential further studies not previously identified.

### Data Extraction

Data were extracted following a preliminary coding protocol shared by all the authors. The extraction of the studies' characteristics included the clinical sample type and demographic features, the study design, the measures of metacognitive beliefs, and if evaluated, the pre- and post-observations of the metacognitive therapy efficacy.

The extraction of the study data also included the aims, hypotheses, and key findings (including the correlation coefficients or the means and standard deviations).

### Quality Assessment

The Newcastle Ottawa Scale (NOS) for quality assessment was employed in this systematic review (www.ohri.ca/programs/clinical_epidemiology/oxford.asp).

Since the majority of the included studies were designed as cross-sectional, we additionally employed an adapted version of the NOS for cross-sectional studies, as previously used (Herzog et al., 2013). The validated NOS for quality assessment was employed for the remaining included studies designed as case-controlled.

Two independent authors (VL, AS) assessed the methodological quality of the retrieved evidence in order to identify any potential source of bias. Disagreements were resolved by consensus with a senior author.

A summary of the quality assessment of included studies is provided in Tables 2, 3.

## Results

### Literature Search Results

**Figure 1:**
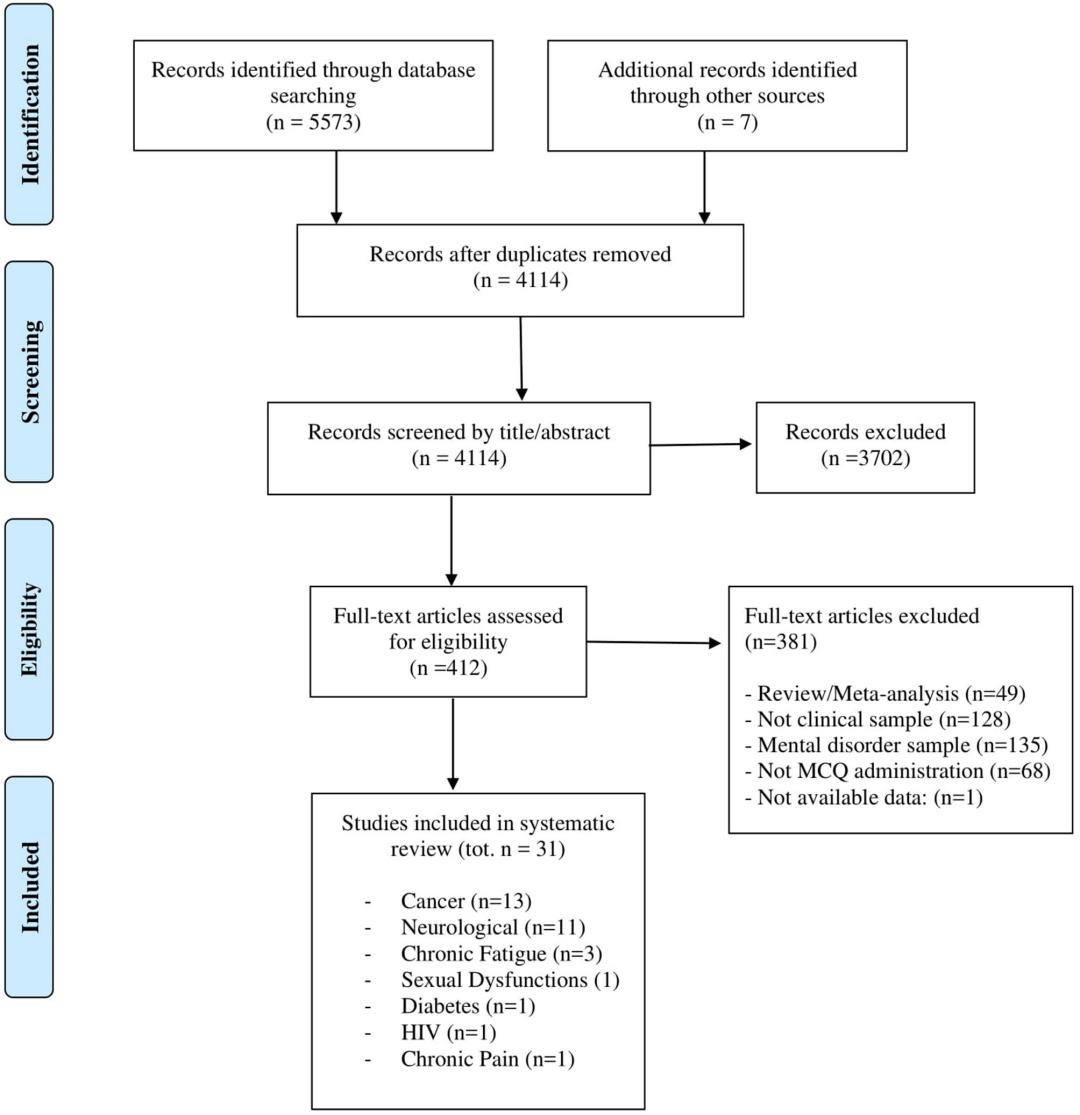
PRISMA 2009 flow diagram. From Moher et al. (2009).

An overview of the screening procedure is provided in Figure 1. The online search strategy retrieved 5,573 papers; a secondary independent manual search retrieved a further seven articles. After removing 1,466 duplicates, 4,114 articles were screened by the title/abstract. A total of 412 full text articles were consequently assessed for eligibility. Finally, 31 studies were included in the systematic review.

### Included Studies

A summary of the included studies' characteristics is provided in Table 1.

**Table 1:** Characteristics of the included studies.

**Study**	**Clinical** **sample**	**Study design**	**MC** **tool**	**Main findings**
Allott et al. (2005)	Parkinson's disease (*n* = 44)	Cross-sectional	MCQ-30	Dysfunctional metacognitive style is independently associated with the increased vulnerability to distress (*p* < 0.05).
Bagcioglu et al. (2012)	Premature ejaculation (*n* = 40) Erectile dysfunction (*n* = 40) Healthy controls (*n* = 80)	Case-controlled Cross-sectional	MCQ-30	The total MCQ-30 score was significantly higher in the patients with premature ejaculation and an erectile dysfunction (*p* < 0.05). The positive beliefs, negative beliefs scores were significantly higher in patients with sexual disorders (*p* < 0.05).The cognitive self-consciousness score was significantly higher in the patients with premature ejaculation, than the erectile dysfunction group (*p* < 0.05), and the healthy controls (*p* < 0.05).
Brown and Fernie (2015)	Parkinson's disease (*n* = 106)	Cross-sectional	MCQ-30	Metacognitive factors were significantly correlated to anxiety when controlling for the motor experiences of daily living and the intolerance of uncertainty, *R*^2^ = 0.56, *F*_(1, 82)_ = 15.04, *p* < 0.001 (adjusted *R*^2^ = 0.53). Metacognitions regarding uncontrollability and danger were significantly correlated to off-period distress when controlling for the motor experiences of daily living, the intolerance of uncertainty, and other metacognitive factors, χ2(1) = 20.52, *p* = 0.001.
Butow et al. (2015)	Breast/prostate cancer (*n* = 63)	Cross-sectional	MCQ-30	Survivors with the clinical fear of cancer recurrence (FCR) had significantly higher positive beliefs about worry (10.1 vs. 7.4, *p =* 0.002) and beliefs about the uncontrollability and dangers of worrying (12.0 vs. 7.7, *p =* 0.000), than those with non-clinical FCR. Total metacognition scores were significantly correlated to FCR in the multiple regression analysis (β = 0.371, *p =* 0.001).
Butow et al. (2017)	Breast/colorectal cancer or Melanoma (Intervention group *n* = 121; Control group *n* = 101)	Randomized controlled trial	MCQ-30 (+MCT)	The efficacy of intervention based on attention training, metacognition, acceptance, screening behavior, and values-based goal setting, compared to the intervention based only on attention control, resulted in the reduction of Fear of Cancer Recurrence Inventory (FCRI) immediately post-therapy, and 3 and 6 months later. The participants also showed a significantly higher T0 to T1 improvement in total MCQ30 (*P* = 0.042) and the need to control thoughts (*P* = 0.004), than controls.
Cook et al. (2014a)	Breast*/*prostate cancer (*n* = 229)	Cross-sectional (validation of MCQ-30 for cancer)	MCQ-30	Confirmatory and exploratory factor analyses supported the validity of the 5-factor structure of the MCQ-30. The structural equation modeling indicated that the two dimensions of metacognition (positive and negative beliefs about worry) were significantly associated to anxiety and depression.
Cook et al. (2014b)	Breast*/*prostate cancer (*n* = 206)	Prospective cohort study	MCQ-30	Metacognitive beliefs (“negative beliefs about worry,” “positive beliefs about worry,” “cognitive confidence”) explained 19% of the variance in anxiety, 15 % of the variance in depression and 14 % of the variance in trauma after 12 months post the cancer diagnosis.
Cook et al. (2015)	Breast*/*prostate cancer (*n* = 229)	Cross-sectional	MCQ-30	Regression analysis showed that metacognitive beliefs were associated to the symptoms of anxiety, depression, and PTSD.
Donnellan et al. (2016)	Post-stroke (*n* = 64)	Cross-sectional	MCQ-30	The total MCQ-30 scores were significantly associated to both, anxiety (*r* = 47, *P* = 0.001) and depression (*r* = 0.54, *p* < 0.0001). The MCQ-30 subscales “cognitive confidence,” “cognitive self-consciousness,” and “uncontrollability/danger” were the specific factors to be associated to the mood symptoms (*p* < 0.01).Metacognition remained a statistically significant factor associated to depression (β = 0.42, *p* < 0.0001) and anxiety (β = 0.51, *p* < 0.0001) after adjusting for education and global cognition.
Fernie et al. (2015)	Chronic Fatigue Syndrome (*n* = 171) (CBT group *n* = 115; GET group *n* = 55)	Cross-sectional Controlled	MCQ-30	The changes on the subscales of the MCQ-30 were found significantly associated to fatigue severity independently of the changes in depression and anxiety, and across the treatment modalities (CBT and GET).
Fisher et al. (2019)	Cancer survivors (*n* = 27)	Open trial (3- and 6 months follow-up)	MCQ-30 (+MCT)	MCT was associated to the statistically significant reductions across all psychological outcomes (anxiety, depression, fear of cancer recurrence, post-traumatic stress symptoms, health-related quality of life, and metacognitive beliefs) which were maintained throughout the 6-month follow-up.
Fisher et al. (2018a)	Cancer survivors (*n* = 87)	Cross-sectional survey	MCQ-30	The MCQ-30 subscales were all positively correlated to the emotional distress and post-traumatic stress symptoms, with the “Negative Beliefs about Worry” subscale strongly correlating to both the distress (*r* = 0.74, *p* < 0.01) and the post-traumatic stress (*r* = 0.70, *p* < 0.01).
Fisher et al. (2018b)	Epilepsy (*n* = 457)	Cross-sectional survey	MCQ-30	The hierarchical regression analyses demonstrated that metacognitive beliefs and illness perceptions were both associated to anxiety and depression when controlling for the influence of demographic variables and epilepsy characteristics. However, metacognitive beliefs accounted for more variance in anxiety and depression, than the illness perceptions.
(Fisher and Noble, 2017)	Epilepsy (*n* = 349)	Cross-sectional survey	MCQ-30	After controlling for demographics, epilepsy characteristics, comorbid physical and/or psychiatric illnesses, metacognitive beliefs explained an additional 20% of the variance in anxiety and 24% additional variance in depression. The relationship between the negative metacognitive beliefs about the uncontrollability and the dangers of worrying and the anxious and depressive symptoms was partially mediated by worry.
Fisher et al. (2017)	Cancer survivors (*n* = 4)	Non-concurrent multiple baseline design with 3- and 6-months follow-up	MCQ-30 (+MCT)	MCT was associated to clinically significant reductions in anxiety, depression, fear of cancer recurrence, worry/rumination, and metacognitive beliefs at the end of treatment, and the gains were maintained in all the patients to the 3 months follow-up and in three out of four patients, to the 6-months follow-up.
Fisher et al. (2016)	Epilepsy (*n* = 349)	Cross-sectional (validation of MCQ-30 for epilepsy)	MCQ-30	The MCQ-30 was found to be a robust measure of metacognitive beliefs and processes and has clinical utility for the subjects with epilepsy.
Fisher et al. (2015)	Adolescent/Young adult Cancer survivors (*n* = 12)	Pilot open trial (6 months follow-up)	MCQ-30 (+MCT)	MCT was associated to the large and statistically significant reductions in anxiety, depression, trauma symptoms, and metacognitive beliefs and processes. In the intention-to-treat sample, 50% of participants met the standardized criteria for recovery on the HADS post-treatment and these gains were maintained through to 6-month follow-up.
Gill et al. (2015)	Traumatic brain injury (*n* = 47) Subarachnoid hemorrhage (*n* = 93)	Cross-sectional	MCQ-30	All the metacognitive variables were positively correlated to PTSS severity: positive metacognitive beliefs about worry (*r* = 0.35, *p* < 0.01), negative metacognitive beliefs about worry (*r* = 0.63, *p* < 0.01) and beliefs about the need to control thoughts (*r* = 0.54, *p* < 0.01). The metacognitive factors were able to explain an additional and significant amount of variance in PTSS severity within the regression analysis.
Heffer-Rahn and Fisher (2018)	Multiple sclerosis (*n* = 132)	Cross-sectional	MCQ-30	Four of the metacognition subscales were positively associated with distress (PMCBS, NMCBS, CC, and NC, *r* = 0.37–0.49, *p* < 0.01). An hierarchical regression predicting distress showed that the metacognitive variables made a significant contribution to the variance in distress, accounting for an additional 4.5% of the variance (F_change_ = 2.695, df = 5,113, *p* < 0.05).
Jacobsen et al. (2016)	Chronic fatigue with subjective cognitive impairment (*n* = 137)	Cross-sectional (pre-post-occupational therapy observation)	MCQ-30	The total MCQ-30 score and the “cognitive confidence” subscale score were significantly associated to the subjective cognitive impairments at the baseline (*p* < 0.0001). The pre-treatment (occupational therapy) scores on “cognitive confidence” were significantly associated with the post-treatment scores on the EMQ (*p* < 0.0001). A hierarchical regression showed that a reduction on MCQ-30 total score was significantly associated to a reduced post- treatment score on the EMQ. Post-treatment scores on “cognitive confidence” were significantly associated with post-treatment scores on the EMQ.
La Foresta et al. (2015)	Amyotrophic Lateral Sclerosis (ALS) patients' caregivers with executive dysfunction (*n* = 22)	Cross-sectional	MCQ-30	The MCQ-30 total score is positively correlated to the number of perseverative errors on the Wisconsin Card Sorting Test (0.75 *p* < 0.001). In particular, the “need to control thoughts” is positively correlated to the number of perseverative errors (0.78 *p* < 0.001).
Maher-Edwards et al. (2011)	Chronic Fatigue Syndrome (*n* = 96)	Cross-sectional	MCQ-30	The correlation analysis showed that metacognitions were positively correlated to the measures of symptom severity, independently of anxiety, and depression. A hierarchical regression analysis indicated that the lack of cognitive confidence was associated to the mental and physical factors of the CFQ, and to the physical functioning independently of the negative emotions. The beliefs about the need to control thoughts was associated to the mental factor of the CFQ independently of the negative emotions and the lack of cognitive confidence.
Mutlu et al. (2018)	Cancer (*n* = 279) Controls (*n* = 212)	Cross-sectional Case controlled	MCQ-30	There was a significant effect in the group on the total MCQ-30 for the two conditions [F_(1, 489)_ = 56.57, *p* = 0.00]. The patients scored significantly higher on all the subscales of the MCQ- 30, compared to the control group. The patients had consistently higher levels of negative metacognitions, compared to the control group regardless of the specific cancer diagnosis.
Purewal and Fisher (2018)	Type1 Diabetes (*n* = 335) Type2 Diabetes (*n* = 279)	Cross-sectional	MCQ-30	The regression analysis showed that metacognitive beliefs were associated to anxiety and depression in the patients with diabetes (PwD) and explained the additional variance in both, anxiety and depression after controlling for demographic variables and illness perceptions.
Quattropani et al. (2018d)	Amyotrophic lateral sclerosis caregivers (*n* = 70)	Cross-sectional	MCQ-30	Metacognition was significantly related to the state and the traits of anxiety, cognitive, and the somatic aspects of depression and the caregiver burden. “Negative beliefs about worry regarding the uncontrollability and danger,” showed the strongest correlations to all the above-mentioned aspects. The negative beliefs showed the strongest correlations to the maladaptive coping strategy.
Quattropani et al. (2018c)	Multiple sclerosis (*n* = 50) Healthy subjects (*n* = 50)	Cross-sectional	MCQ-30	The *T*-test for two independent samples (using the Bonferroni correction) showed insignificant differences for metacognitions between the MS patients and the healthy subjects. A positive and moderate correlation was found between “cognitive confidence” and depression for the MS patients, but not for the control subjects. The negative beliefs were positively correlated to depression in the MS patients, but not in the control subjects. Moderate positive correlation between “cognitive self-consciousness” and depression was observed in the control subjects, but not in the MS patients.
Quattropani et al. (2017a)	Breast cancer subjects undergoing chemotherapy (*n* = 80)	Cross-sectional	MCQ-30	The results of the regression analysis has shown that the negative beliefs were significantly associated to anxiety, depression and overall distress.
Quattropani et al. (2016)	Cancer patients undergoing chemotherapy (*n* = 175)	Cross-sectional	MCQ-30	The negative beliefs had the strongest correlation to both anxiety (*r* = 0.74; *p* < 0.01) and depression (*r* = 0.58; *p* < 0.01). The cognitive confidence showed low correlation coefficients to anxiety (*r* = 0.24; *p* < 0.01) and depression (*r* = 0.22; *p* < 0.01). The positive beliefs had a low significant correlation to anxiety (*r* = 0.20; *p* < 0.05), but not to depression. The total score of the MCQ was positively related to all the other observed variables.
Strodl et al. (2015)	HIV community subjects (*n* = 106)	Cross-sectional	MCQ-30	The Negative Metacognitive Beliefs was the only metacognitive beliefs involved in the relationship between the HIV stigma perceptions and the depressive and anxious symptoms.
Toffalini et al. (2015)	Parents of children with cancer (*n* = 30) Hospitalized control parents (*n* = 36) Healthy control parents (*n* = 30)	Cross-sectional Controlled	MCQ-30	The parents in both the study group and the hospitalized control group reported less SWB than the healthy control group. Metacognition explained up to 77% of the variance in the SWB in the parents of children with cancer, compared to only 23% in the hospitalized control group and 33% in the healthy control group.
Ziadni et al. (2018)	Chronic pain (*n* = 211)	Cross-sectional (2 weeks)	MCQ-30	The participants with higher average levels of daily pain intensity and the negative metacognitive beliefs about worry reported higher levels of daily pain catastrophizing, as well as daily depression, and anxiety. Some aspects of the metacognitive beliefs (i.e., dangerousness and the uncontrollability of thoughts) were also negatively associated to the average daily levels of positive effects. However, these effects were not interactive; the metacognitive beliefs did not moderate the relationships of pain catastrophizing with the other daily variables.

**Table 2:** Quality assessment of included cross sectional studies.

**Study**	**Selection**	**Comprability**	**Outcome**	**NOS stars**
Allott et al. (2005)	***	*	*	5
Brown and Fernie (2015)	***	**	*	6
Butow et al. (2015)	***	**	*	6
Cook et al. (2014a)	***	**	*	6
Cook et al. (2014b)	***	**	*	6
Cook et al. (2015)	***	**	*	6
Donnellan et al. (2016)	***	**	*	6
Fisher et al. (2018a)	****	**	*	6
Fisher et al. (2015)	***	**	*	6
Fisher et al. (2017)	***	**	*	6
Fisher et al. (2016)	***	**	*	6
Gill et al. (2015)	***	**	*	6
Heffer-Rahn and Fisher (2018)	**	**	*	5
Jacobsen et al. (2016)	**	**	*	5
La Foresta et al. (2015)	***	**	*	6
Maher-Edwards et al. (2011)	***	**	*	6
Purewal and Fisher (2018)	**	**	*	5
Quattropani et al. (2018d)	***	**	*	6
Quattropani et al. (2018c)	***	**	*	6
Quattropani et al. (2017a)	***	*	*	5
Quattropani et al. (2016)	***	**	*	6
Strodl et al. (2015)	***	**	*	6
Ziadni et al. (2018)	***	**	*	6

*NOS, Newcastle Ottawa Scale. An adapted NOS for cross-sectional was employed. Each study can be awarded of a maximum of ten NOS stars*.

**Table 3:** Quality assessment of included case-controlled studies.

**Study**	**Selection**	**Comprability**	**Exposure**	**NOS stars**
Bagcioglu et al. (2012)	***	*	**	6
Fernie et al. (2015)	**	**	**	6
Mutlu et al. (2018)	***	**	**	7
Toffalini et al. (2015)	***	**	**	7

*NOS, Newcastle Ottawa Scale. Each study can be awarded of a maximum of nine NOS stars*.

The majority of included studies were designed as cross-sectional. A limited number of studies involved a control group.

The majority of the studies involved patients with cancer or different neurological diseases. Studies on Chronic Fatigue Syndrome, Diabetes, Sexual Dysfunctions, Chronic Pain, and HIV were also retrieved and included in the systematic review.

#### Metacognition and Cancer

The potential role of metacognitive beliefs in subjects with cancer has been a debated research topic in the last few years. The MCQ-30 has been recently validated in a primary breast/prostate cancer population and it is actually considered a valid measure of metacognition among these patients (Cook et al., 2014a); subjects with cancer were found to exhibit more negative metacognitive beliefs compared to healthy subjects (Mutlu et al., 2018).

Metacognitive beliefs are also associated with the symptoms of anxiety, depression, and post-traumatic stress disorder (PTSD), and they independently explain the additional variance in these outcomes even after controlling for demographic characteristics and illness perceptions (Cook et al., 2014b, 2015). Moreover, it was recently discussed that negative beliefs highly correlated to anxiety and depression, and were also independently associated with anxiety in subjects undergoing chemotherapy (Quattropani et al., 2016, 2017a).

Patients experiencing the fear of cancer recurrence (FCR), often show dysfunctional metacognitive beliefs, particularly positive beliefs about worry and beliefs about the uncontrollability and dangers of worrying (Butow et al., 2015). In a recent randomized controlled trial (RCT), the efficacy of a multidimensional intervention, based on metacognition training, in reducing the fear of cancer recurrence was demonstrated. At the end of the trial, subjects also exhibited lower maladaptive metacognitive beliefs (Butow et al., 2017).

The potential positive role of metacognition therapy (MCT) has also been investigated in young cancer survivors who commonly show signs of dysfunctional metacognitive beliefs associated with emotional distress and post-traumatic stress symptoms (PTSS) (Fisher et al., 2018a): MCT was shown to be effective in reducing anxiety and depression symptoms (Fisher et al., 2015).

The MCT sessions appear as having a positive effect even on reducing the levels of anxiety, depression, FCR, post-traumatic stress symptoms, health-related quality of life, worry/rumination, and maladaptive metacognitive beliefs in cancer survivors (Fisher et al., 2017, 2019).

We retrieved only one study that investigated the impact of metacognition in parents of children with cancer, suggesting that metacognitive beliefs might be related to the development of psychological distress and emotional disorders not only in cancer patients but also in caregivers (Toffalini et al., 2015). The authors showed that the parents of such children exhibited worse self-well-being compared to the control parents. Specifically, the dysfunctional metacognitive factors explained a higher variance of self-well-being in the parents of children with cancer, compared to the parents of healthy children, as well as the parents of hospitalized children.

#### Metacognition in Post-stroke Patients

The connection between metacognition and mood has been broadly investigated in several medical conditions related to psychological comorbidity. However, the study conducted by Donnellan et al. (2016) represents the first attempt to describe this connection in post-stroke patients. Patients experiencing post-stroke anxiety and depression symptoms also show stronger metacognitive beliefs regarding cognitive confidence, cognitive self-consciousness, uncontrollability, and danger. According to the authors' conclusions, this metacognitive profile may affect both the patient's cognitive processing and actions, consequently worsening the psychological distress.

The MCQ-30 proved to be a valuable tool in assessing the metacognition in a stroke cohort, even though the small sample size did not permit further examinations.

#### Metacognition and Parkinson's Disease

Parkinson's disease (PD) is frequently associated with several psychological burdens, often shared between patients and their caregivers. The hypothesis stating that metacognitive beliefs may affect the modalities of how patients adapt to the disease has been recently tested.

The dysfunctional metacognitive style was found to be independently associated with increased distress in patients with PD (Allott et al., 2005). Moreover, the authors suggested that subjects who exhibit stronger negative beliefs about worrying may report elevated levels of distress.

Further considerations about the role of metacognition in PD have been recently discussed by Brown and Fernie (2015). The authors confirmed that dysfunctional metacognitive beliefs are associated with the worsening of anxiety levels in PD patients. They also suggested, for the first time, that metacognition might be also independently associated with the development of the off period distress.

#### Metacognition and Chronic Fatigue Syndrome (CFS)

Several studies have previously investigated the impact of psychological factors in subjects affected by CFS, highlighting that anxiety, depression, and stress are commonly associated with this condition (Afari and Buchwald, 2003). The potential role of metacognition has also been investigated owing to its known association with negative emotions.

Metacognitive beliefs, particularly negative beliefs about thoughts regarding uncontrollability, cognitive confidence, and beliefs about the need to control one's thoughts, might be independently correlated to symptom severity in CFS, regardless of the negative emotions (Maher-Edwards et al., 2011). In this cross-sectional study, the authors for the first time showed that metacognition was a better independent factor associated with physical and psychological symptom severity than anxiety and depression.

Metacognitive beliefs were also recently found to have a significant effect on fatigue severity even across the commonly used treatment modalities, such as cognitive behavioral therapy (CBT) and graded exercise therapy (GET). The authors discussed that the relation between metacognition and fatigue could be mediated by CBT, since metacognition is an indirectly addressed variable in CBT programs. On the other hand, they suggested that the relationship between metacognitive beliefs and the changes in fatigue severity might reflect decreased worry and symptom pre-occupation, which are variables that have been shown to mediate the outcomes in GET (Fernie et al., 2015).

Another recent study reported for the first time the associations between dysfunctional metacognitive beliefs and subjective cognitive impairments in CFS patients (Jacobsen et al., 2016). Specifically, the baseline scores on the subscale of cognitive confidence were independently associated with the subjective cognitive impairment at the end of an occupational therapy-based intervention (Return-To-Work, RTW). Moreover, a reduction in dysfunctional cognitive confidence while undergoing treatment was found to be associated with less subjective cognitive impairments at the end of the RTW intervention.

#### Metacognition and Epilepsy

The investigation of metacognition in subjects affected by epilepsy was aimed at achieving a better conceptualization of the psychological mechanisms that contribute to anxiety and depression, which are commonly related to this condition. The MCQ-30 was previously validated as a valuable measure of metacognitive beliefs, with a substantial clinical utility within subjects with epilepsy (Fisher et al., 2016).

The potential role of the metacognition model in explaining anxiety and depression in subjects suffering from epilepsy was recently investigated for the first time (Fisher and Noble, 2017). The authors showed that negative metacognitive beliefs about the uncontrollability and the dangers of worrying were independently associated with the symptoms of anxiety and depression. Furthermore, it was highlighted that metacognitions also explained additional variance in anxiety and depression independently of demographic characteristics, epilepsy-related variables, and the patients' illness perception (Fisher et al., 2018b).

#### Metacognition and Acquired Brain Injury (ABI)

Metacognition was recently investigated as a potential mediator of PTSS severity in individuals with Acquired Brain Injury (ABI) (Gill et al., 2015). Authors supported the application of a novel metacognitive model of PTSD for those with an ABI. The study highlighted that the negative beliefs about the uncontrollability and the dangers of worrying, as well as the beliefs about the need to control one's thoughts, were independently associated with the PTSS severity in subjects after a brain injury.

#### Metacognition and Multiple Sclerosis (MS)

Metacognitive beliefs were previously associated with emotional distress in neurological conditions. A recent study (Heffer-Rahn and Fisher, 2018) for the first time investigated the potential role of metacognitive beliefs in patients with MS. The authors showed that the positive beliefs about worry, the negative beliefs about the uncontrollability and the dangerous nature of worry, the cognitive confidence, and the need to control one's thoughts were positively associated with distress. Furthermore, metacognition independently explained the additional variance in the distress of the patients with MS.

Some interesting results have been discussed in an equally recent case-controlled study (Quattropani et al., 2018c) aimed at examining the relationships between metacognition, anxiety, and depression in MS patients and healthy subjects. This is the first controlled study exploring metacognition in MS patients. The authors indicated that patients and healthy controls showed no significant differences in terms of metacognitive beliefs. According to an authors' discussion, a suggested role of metacognitions as vulnerability factors in predicting the development of psychological symptoms would explain their findings, even though further investigation would be needed.

Moreover, the cognitive confidence, positive beliefs, negative beliefs, and the need to control one's thoughts were positively correlated to anxiety and the overall distress in both MS patients and healthy subjects. However, the association between these metacognitive factors and depression was conflicting in patients and controls, with the positive correlations only seen in the patients. According to the authors, these evidence might suggest a different impact of metacognition on the distress variables between the MS patients and healthy subjects.

#### Metacognition and Amyotrophic Lateral Sclerosis (ALS)

The role of metacognitive factors among caregivers is a research topic not usually investigated, at least in the context of medical conditions. In this regard, the assessment of metacognition in the ALS patients' caregivers represents a recently explored field.

Our systematic search retrieved the first multicentric study (Quattropani et al., 2018d) which indicated dysfunctional metacognitive beliefs (namely, negative beliefs about worry, about uncontrollability, and danger) as being significantly related to state and trait anxiety, depression, and to the status of burden in the ALS patients' caregivers. The authors highlighted the relevance of exploring metacognitive factors in caregivers in order to identify profiles potentially at risk of developing distress and other burden-related symptoms.

Metacognitive beliefs have also been studied in ALS patients' caregivers as factors potentially involved in executive functions (EF) regulation, since caregiving requires abilities such as cognitive flexibility, self-regulation, and self-consciousness, which are commonly related to both metacognitive processes and executive functioning (La Foresta et al., 2015). The authors effectively discussed the relationship between metacognitive factors and perseveration exhibited on the Wisconsin Card Sorting Test, used to assess EF. According to the authors, this finding suggests that the tendency to perseverate could be closely linked to dysfunctional metacognitive beliefs, as an expression of a specific inflexibility in thinking processes.

#### Metacognition and Diabetes

Only one study investigating metacognitive beliefs in subjects with diabetes met the required inclusion criteria for our systematic review. Anxiety and depression are common in people with diabetes. According to Purewal and Fisher (2018), metacognition is a factor capable of explaining anxiety and depression, independently of demographic characteristics and illness perception. The negative beliefs about the uncontrollability and the dangers of worrying, and a lack of cognitive confidence, appeared to be the most significant metacognitive factors associated with anxiety and depression.

#### Metacognition and Chronic Pain

Metacognitive beliefs have recently been indicated as factors independently associated with pain and its impact on daily functioning among subjects suffering from Chronic Pain (CP) (Ziadni et al., 2018). Particularly, the authors showed that the metacognitive beliefs about the uncontrollability and the danger of thoughts, as well as those related to self-consciousness, were independently related to the daily levels of psychological functioning. However, none of the metacognitive factors were able to modify the intensity of the relationships between pain catastrophizing and emotional conditions. Therefore, the authors suggest that metacognitive beliefs might be considered as an index of poor psychological adjustment to chronic pain, rather than a risk factor which amplifies the immediate negative consequences of catastrophizing.

#### Metacognition, HIV, and Sexual Dysfunction

The investigation of the potential role of metacognition eventually included HIV and Sexual Dysfunctions.

Subjects with HIV show stronger negative metacognitive beliefs, which are significantly associated with their anxiety and depression symptoms, and to the increased psychological distress resulting from the disease-related stigma (Strodl et al., 2015).

Patients with sexual dysfunctions seem to focus on the metacognitive belief that worrying could have positive effects in solving problems and avoiding unpleasant situations which are associated with sexual disorders (Bagcioglu et al., 2012).

## Discussion

### Summary of Main Findings

As per our understanding, this is the first attempt to systematically review the studies aimed at investigating metacognitive beliefs in patients with chronic medical conditions and their caregivers. Particularly, the dysfunctional metacognitive factors according to the S-REF model and their relationships with both emotional and psychological distress were investigated.

We have focused our research on metacognition as postulated by Wells, who emphasized how metacognitive processes might incline individuals toward developing response patterns to perceived cognitive, behavioral, or emotional difficulties (Wells, 2000b). In light of this perspective, the Metacognitions Questionnaire (MCQ) and its short version (MCQ-30) have been considered as reliable measures of metacognitive beliefs and processes.

Metacognition has been originally defined as, “the aspect of information processing that monitors, interprets, evaluates, and regulates the contents and processes of its organization.” (Wells and Purdon, 1999). Owing to its association to the development and maintenance of psychological dysfunctions, metacognitive beliefs have been broadly investigated in the context of both non-clinical (Spada et al., 2008b) and psychiatric samples (Sellers et al., 2016; Sun et al., 2017).

In recent decades, the study of metacognition has also extended to patients suffering from different medical conditions, which negatively affected their quality of life and exposed them to psychological distress.

The studies included in this systematic review have mostly considered different chronic medical conditions, such as cancer and neurological diseases. Metacognition has been specifically explored in those medical conditions which are often related to increased anxiety and depression, with negative effects on patient's quality of life. The majority of these diseases seem to exhibit a metacognitive profile mainly characterized by the presence of negative beliefs factors, which have been described as significantly associated with emotional and psychological distress. In this regard, negative beliefs about worry, its uncontrollability, and dangers seem to represent a common metacognitive pattern across the investigated pathologies, even when motor dysfunctions are involved (as in Parkinson's off-periods).

In the context of the investigated medical conditions, it has been shown that living with cancer and surviving cancer might lead to the development of dysfunctional negative beliefs about a patient's future, as well as to the development of PTSS. In this context, MCT has been described as a valid intervention that reduces both the emotional distress and the fear of recurrence in cancer survivors.

In addition to the evidenced general pre-disposition to exhibit negative beliefs, cognitive confidence has been described as another relevant factor. It is a measure of an individual's confidence in his own attention and memory. It has been found being associated with psychological and emotional distress, and it might negatively affect coping strategies when the patient feels mentally fatigued (as in neurological conditions, cancer, or chronic fatigue).

Finally, an important issue is the role of metacognition among caregivers of subjects affected by a chronic illness. The patients' caregivers are often exposed to a high risk of emotional and psychological burden, particularly in relation to the degree of the patient's behavioral or physical impairment. However, only two studies have been retrieved investigating the metacognition in caregivers, and they specifically involved the parents of children with cancer and the caregivers of ALS patients. In this context, metacognitive beliefs seem to be involved in emotional distress even in caregivers. Further studies offering an insight on the role of metacognitive beliefs in caregivers are needed in order to better characterize psychological distress in caregivers.

In this regard, a recent study on a sample of health care professionals provided evidence on the differential role of metacognitions in predicting the risk of burnout (Quattropani et al., 2017b).

### Limitations

While included studies showed an adequate quality as evidenced through the NOS assessment, most of them presented some methodological weaknesses.

The majority of studies adopted a cross-sectional design, which does not allow for inferences of causality. Furthermore, the majority of the reviewed studies did not involve a control sample or other conditions for comparison. In addition, the absence of a control sample could make it difficult to attribute the findings to that specific medical condition.

Finally, only a limited number of studies reported temporal variations of metacognitive factors evaluation, adopting follow-up observations. Longitudinal studies are strongly needed and recommended in order to deepen understanding of the impact of dysfunctional metacognitive beliefs on psychological and emotional distress. Additionally, longitudinal observations would better clarify the potential efficacy of psychological intervention based on metacognition.

### Conclusions

The findings of this systematic review provide evidence that dysfunctional metacognitive beliefs are significantly associated with emotional and psychological distress in patients with medical chronic conditions, and caregivers. Therefore, within the compendium of psychological assessments usually performed in the context of medical chronic conditions, even the additional evaluation of metacognitive factors appears valuable.

Based on the evidenced association between metacognition and negative emotions, psychological interventions centered on the metacognitive approach (Wells, 2000b) could have positive effects on emotional and psychological distress in patients with chronic medical conditions, and their caregivers.

## Data Availability Statement

The datasets generated for this study are available on request to the corresponding author

## Author Contributions

MQ and VL contributed to the conception of this systematic review. VL and AS performed the literature search and wrote the first draft of the manuscript. MQ and GM revised the first draft of the manuscript. All authors contributed to the subsequent drafting and rewriting of the manuscript and approved the final version of the manuscript.

### Conflict of Interest

The authors declare that the research was conducted in the absence of any commercial or financial relationships that could be construed as a potential conflict of interest.

## References

[B1] Afari N. Buchwald D. (2003). Chronic fatigue syndrome: a review. Am. J. Psychiatry. 160, 221–236. 10.1176/appi.ajp.160.2.221 12562565

[B2] Allott R. Wells A. Morrison A. P. Walker R. (2005). Distress in Parkinson's disease: contributions of disease factors and metacognitive style. Br. J. Psychiatry 187, 182–183. 10.1192/bjp.187.2.182 16055832

[B3] American Psychiatric Association (2013). DSM-5. Diagnostic and Statistical Manual of Mental Disorders, 5th Edn. Washington, DC: American Psychiatric Association.

[B4] Bagcioglu E. Altunoluk B. Bez Y. Soylemez H. Asik A. Emul M. (2012). Metacognition in patients with premature ejaculation and erectile dysfunction. J. Cogn. Behav. Psychother. 12, 77–84.

[B5] Brown R. G. Fernie B. A. (2015). Metacognitions, anxiety, and distress related to motor fluctuations in Parkinson's disease. J. Psychosom. Res. 78, 143–148. 10.1016/j.jpsychores.2014.09.021 25311871

[B6] Butow P. Kelly S. Thewes B. Hruby G. Sharpe L. Beith J. (2015). Attentional bias and metacognitions in cancer survivors with high fear of cancer recurrence. Psychooncology 24, 416–423. 10.1002/pon.3659 25156065

[B7] Butow P. N. Turner J. Gilchrist J. Sharpe L. Smith A. B. Fardell J. E.. (2017). Randomized trial of conquerfear: A novel theoretically based psychosocial intervention for fear of cancer recurrence. J. Clin. Oncol. 35, 4066–4077. 10.1200/JCO.2017.73.1257 29095681

[B8] Cartwright-Hatton S. Wells A. (1997). Beliefs about worry and intrusions: the meta-cognitions questionnaire and its correlates. J. Anxiety Disord. 11, 279–296. 10.1016/S0887-6185(97)00011-X 9220301

[B9] Catalano A. Martino G. Bellone F. Gaudio A. Lasco C. Langher V.. (2018). Anxiety levels predict fracture risk in postmenopausal women assessed for osteoporosis. Menopause 25, 1–6. 10.1097/GME.0000000000001123 29738418

[B10] Cook S. A. Salmon P. Dunn G. Fisher P. (2014a). Measuring metacognition in cancer: validation of the metacognitions questionnaire 30 (MCQ-30). PLoS ONE 9:e107302. 10.1371/journal.pone.0107302 25215527 PMC4162595

[B11] Cook S. A. Salmon P. Dunn G. Holcombe C. Cornford P. Fisher P. (2014b). A prospective study of the association of metacognitive beliefs and processes with persistent emotional distress after diagnosis of cancer. Cognit. Ther. Res. 39, 51–60. 10.1007/s10608-014-9640-x PMC4312385 25657483

[B12] Cook S. A. Salmon P. Dunn G. Holcombe C. Cornford P. Fisher P. (2015). The association of metacognitive beliefs with emotional distress after diagnosis of cancer. Health Psychol. 34, 207–215. 10.1037/hea0000096 25133826 PMC4321533

[B13] Cooper M. J. Grocutt E. Deepak K. Bailey E. (2007). Metacognition in anorexia nervosa, dieting and non-dieting controls: a preliminary investigation. Br. J. Clin. Psychol. 46, 113–117. 10.1348/014466506X115245 17472205

[B14] De Luca Picione R. Martino M. L. Freda M. F. (2017). Modal articulation: the psychological and semiotic functions of modalities in the sensemaking process. Theor. Psychol. 28, 84–103. 10.1177/0959354317743580

[B15] Dicé F. Dolce P. Freda M. F. (2016). Exploring emotions and the shared decision-making process in pediatric primary care. Medit. J. Clinical Psychol. 4, 1–31. 10.6092/2282-1619/2016.4.1312

[B16] Dicé F. Dolce P. Maiello A. Freda M. F. (2019). Exploring emotions in dialog between health provider, parent and child. An observational study in pediatric primary care. Prat. Psychol. 10.1016/j.prps.2018.12.001. [Epub ahead of print].

[B17] Dicé F. Santaniello A. Gerardi F. Paoletti A. Valerio P. Freda M. F. (2018). Gli interventi assistiti dagli animali come processi di promozione della salute. Una review sistematica. Psicol. Sal. 3, 5–23. 10.3280/PDS2018-003001

[B18] Donnellan C. Al Banna M. Redha N. Al Sharoqi I. Al-Jishi A. Bakhiet M. (2016). Association between metacognition and mood symptoms poststroke. J. Geriatr. Psychiatry. Neurol. 29, 212–220. 10.1177/0891988716640374 27056067

[B19] Fantinelli S. Marchetti D. Verrocchio M. C. Franzago M. Fulcheri M. Vitacolonna E. (2019). Assessment of psychological dimensions in telemedicine care for gestational diabetes mellitus: a systematic review of qualitative and quantitative studies. Front. Psychol. 10:153. 10.3389/fpsyg.2019.00153 30804842 PMC6370698

[B20] Fernie B. A. Murphy G. Wells A. Nikcevic A. V. Spada M. M. (2015). Treatment outcome and metacognitive change in CBT and GET for chronic fatigue syndrome. Behav. Cogn. Psychother. 44, 397–409. 10.1017/S135246581500017X 25895437

[B21] Fisher P. L. Byrne A. Fairburn L. Ullmer H. Abbey G. Salmon P. (2019). Brief metacognitive therapy for emotional distress in adult cancer survivors. Front. Psychol. 10:162. 10.3389/fpsyg.2019.00162 30766505 PMC6365419

[B22] Fisher P. L. Byrne A. Salmon P. (2017). Metacognitive therapy for emotional distress in adult cancer survivors: a case series. Cognit. Ther. Res. 41, 891–901. 10.1007/s10608-017-9862-9 PMC5656708 29104332

[B23] Fisher P. L. Cook S. A. Noble A. (2016). Clinical utility of the metacognitions questionnaire 30 in people with epilepsy. Epilepsy Behav. 57, 185–191. 10.1016/j.yebeh.2016.02.004 26970994

[B24] Fisher P. L. McNicol K. Cherry M. G. Young B. Smith E. Abbey G. (2018a). The association of metacognitive beliefs with emotional distress and trauma symptoms in adolescent and young adult survivors of cancer. J. Psychosoc. Oncol. 36, 545–556. 10.1080/07347332.2018.1440276 29611779

[B25] Fisher P. L. McNicol K. Young B. Smith E. Salmon P. (2015). Alleviating emotional distress in adolescent and young adult cancer survivors: An open trial of metacognitive therapy. J. Adolesc. Young Adult Oncol. 4, 64–69. 10.1089/jayao.2014.0046 26812553

[B26] Fisher P. L. Noble A. J. (2017). Anxiety and depression in people with epilepsy: the contribution of metacognitive beliefs. Seizure 50, 153–159. 10.1016/j.seizure.2017.06.012 28667910

[B27] Fisher P. L. Reilly J. Noble A. (2018b). Metacognitive beliefs and illness perceptions are associated with emotional distress in people with epilepsy. Epilepsy Behav. 86, 9–14. 10.1016/j.yebeh.2018.07.008 30036766

[B28] García-Montes J. M. Cangas A. Pérez-Álvarez M. Fidalgo Á. M. Gutiérrez O. (2006). The role of meta-cognitions and thought control techniques in predisposition to auditory and visual hallucinations. Br. J. Clin. Psychol. 45, 309–317. 10.1348/014466505X66755 17147098

[B29] Gill I. J. Mullin S. Simpson J. (2015). Are metacognitive processes associated with posttraumatic stress symptom severity following acquired brain injury? Disabil. Rehabil. 37, 692–700. 10.3109/09638288.2014.939774 25019599

[B30] Heffer-Rahn P. Fisher P. L. (2018). The clinical utility of metacognitive beliefs and processes in emotional distress in people with multiple sclerosis. J. Psychosom. Res. 104, 88–94. 10.1016/j.jpsychores.2017.11.014 29275790

[B31] Herzog R. Alvarez-Pasquin J. Diaz C. Del Barrio J. L. Estrada J. E. Gil A. (2013). Are healthcare workers' intentions to vaccinate related to their knowledge, beliefs and attitudes? a systematic review. BMC Public Health 13, 154. 10.1186/1471-2458-13-154 23421987 PMC3602084

[B32] Jacobsen H. B. Aasvik J. K. Borchgrevink P. C. Landro N. I. Stiles T. C. (2016). Metacognitions are associated with subjective memory problems in individuals on sick leave due to chronic fatigue. Front. Psychol. 7:729. 10.3389/fpsyg.2016.00729 27242634 PMC4866616

[B33] Knowles M. M. Foden P. El-Deredy W. Wells A. (2016). A Systematic review of efficacy of the attention training technique in clinical and nonclinical samples. J. Clin. Psychol. 72, 999–1025. 10.1002/jclp.22312 27129094

[B34] La Foresta S. Messina S. Faraone C. Pistorino G. Vita G. Lunetta C. (2015). Conceptualizing the relations between metacognition and executive functions in amyotrophic lateral sclerosis (ALS) patients' caregivers. A preliminary study. Medit. J. Clinical Psychol. 3 10.6092/2282-1619/2015.3.1121

[B35] Larøi F. Linden M. Marczewski P. (2004). The effects of emotional salience, cognitive effort and meta-cognitive beliefs on a reality monitoring task in hallucination-prone subjects. Br. J. Clin. Psychol. 43, 221–233. 10.1348/0144665031752970 15333229

[B36] Lenzo V. Geraci A. Filastro A. Quattropani M. C. (2019). Effect on post-stroke anxiety and depression of an early neuropsychological and behavioural treatment. J. Psychopathol. 25, 63–69.

[B37] Liberati A. Altman D. G. Tetzlaff J. Mulrow C. Gøtzsche P. C. Ioannidis J. P. (2009). The PRISMA statement for reporting systematic reviews and meta-analyses of studies that evaluate healthcare interventions: explanation and elaboration. BMJ. 21:339 10.1136/bmj.b2700 PMC2714672 19622552

[B38] Maher-Edwards L. Fernie B. A. Murphy G. Wells A. Spada M. M. (2011). Metacognitions and negative emotions as predictors of symptom severity in chronic fatigue syndrome. J. Psychosom. Res. 70, 311–317. 10.1016/j.jpsychores.2010.09.016 21414450

[B39] Marchetti D. Carrozzino D. Fraticelli F. Fulcheri M. Vitacolonna E. (2017). Quality of life in women with gestational diabetes mellitus: a systematic review. J. Diabetes Res. 2017:7058082. 10.1155/2017/7058082 28326332 PMC5343261

[B40] Marchini F. Caputo A. Napoli A. Balonan J. T. Martino G. Nannini V. (2018). Chronic illness as loss of good self: underlying mechanisms affecting diabetes adaptation. Medit. J. Clinical Psychol. 6, 1–25. 10.6092/2282-1619/2018.6.1981

[B41] Martino G. Bellone F. Langher V. Caputo A. Catalano A. Quattropani M. (2019c). Alexithymia and psychological distress affect perceived quality of life in patients with type 2 diabetes mellitus. Medit. J. Clinical Psychol. 10.6092/2282-1619/2019.7.2328. [Epub ahead of print].

[B42] Martino G. Catalano A. Bellone F. Russo G. T. Vicario C. M. Lasco A.. (2019d). As time goes by: anxiety negatively affects the perceived quality of life in patients with type 2 diabetes of long duration. Front. Psychol. 10:1779. 10.3389/fpsyg.2019.01779 31428028 PMC6689992

[B43] Martino G. Catalano A. Bellone F. Sardella A. Lasco C. Capr,ì T. (2018). Vitamin D status is associated with anxiety levels in postmenopausal women evaluated for osteoporosis. Medit. J. Clinical Psychol. 6, 1–16. 10.6092/2282-1619/2018.6.1740

[B44] Martino G. Sardella A. Bellone F. Lasco G. Langher V. Cazzato V. (2019e). Executive functions and bone health: a focus on cognitive impulsivity and bone mineral density. Medit. J. Clinical Psychol. 10.6092/2282-1619/2019.7.2167. [Epub ahead of print].

[B45] Martino M. L. Freda M. F. (2016). Meaning-making process related to temporality during breast cancer traumatic experience: the clinical use of narrative to promote a new continuity of life. Eur. J. Psychol. 12, 622–634. 10.5964/ejop.v12i4.1150 27872670 PMC5114876

[B46] Martino M. L. Gargiulo A. Lemmo D. Margherita G. (2019a). Cancer blog narratives: the experience of under-fifty women with breast cancer during different times after diagnosis. Qual. Rep. 24, 158–173.

[B47] Martino M. L. Lemmo D. Gargiulo A. Barberio D. Abate V. Avino F. (2019b). Underfifty women and breast cancer: narrative markers of meaning-making in traumatic experience. Front. Psychol. 10:618 10.3389/fpsyg.2019.00618 30984067 PMC6448035

[B48] Moher D. Liberati A. Tetzlaff J. Altman D. G. PRISMA Group (2009). Preferred reporting items for systematic reviews and meta-analyses: the PRISMA statement. PLoS Med. 6:e1000097 10.1371/journal.pmed.1000097 19621072 PMC2707599

[B49] Mutlu H. H. Bilican F. I. Mutlu H. H. Gumus M. (2018). A comparison of metacognitive factors among patients with cancer and the control group. Psychooncology 27, 1277–1283. 10.1002/pon.4667 29466609

[B50] Normann N. Morina N. (2018). The efficacy of metacognitive therapy: A systematic review and meta-analysis. Front. Psychol. 9:2211 10.3389/fpsyg.2018.02211 30487770 PMC6246690

[B51] Pöttgen J. Lau S. Penner I. Heesen C. Moritz S. (2015). Managing neuropsychological impairment in multiple sclerosis. Int. J. MS. Care 17, 130–137. 10.7224/1537-2073.2014-015 26052258 PMC4455865

[B52] Purewal R. Fisher P. L. (2018). The contribution of illness perceptions and metacognitive beliefs to anxiety and depression in adults with diabetes. Diabetes Res. Clin. Pract. 136, 16–22. 10.1016/j.diabres.2017.11.029 29203257

[B53] Quattropani M. C. Geraci A. Lenzo V. Delle Chiaie R. Filastro A. (2018a). Post stroke anxiety and depression: relationships to cognitive rehabilitation outcome. Clin. Neuropsychy. J. Treat Eval. 15, 12–18.

[B54] Quattropani M. C. La Foresta S. Russo M. Faraone C. Pistorino G. Lenzo V. (2018d). Emotional burden and coping strategies in amyotrophic lateral sclerosis caregivers: The role of metacognitions. Minerva Psichiatr. 59, 95–104. 10.23736/S0391-1772.18.01961-1

[B55] Quattropani M. C. Lenzo V. Armieri V. Filastro A. (2018b). The origin of depression in Alzheimer disease: a systematic review. Riv. Psichiatr. 53, 18–30. 10.1708/2866.28920 29493651

[B56] Quattropani M. C. Lenzo V. Baio M. Bordino V. German,à A. Grasso D. (2017b). Credenze metacognitive e strategie di coping in operatori di cure domiciliari a rischio di burnout. Psicol. Sal. 2, 121–142. 10.3280/PDS2017-002006

[B57] Quattropani M. C. Lenzo V. Filastro A. (2017a). Predictive factors of anxiety and depression symptoms in patients with breast cancer undergoing chemotherapy. An explorative study based on metacognitions. J. Psychopathol. 23, 67–73.

[B58] Quattropani M. C. Lenzo V. Filastro A. (2018c). The role of metacognition in multiple sclerosis: a clinical study and assessment of possible correlation with anxiety, depression and coping strategies. Euromedit. Biomed. J. 9, 39–45.

[B59] Quattropani M. C. Lenzo V. Mucciardi M. Toffle M. E. (2015). Psychometric properties of the Italian version of the short form of the metacognitions questionnaire (MCQ-30). BPA Appl. Psychol. Bull. 269, 30–42.

[B60] Quattropani M. C. Lenzo V. Mucciardi M. Toffle M. E. (2016). Metacognition as predictor of emotional distress in cancer patients. Life Span Disab. 19, 221–239.

[B61] Sellers R. Varese F. Wells A. Morrison A. P. (2016). A meta-analysis of metacognitive beliefs as implicated in the self-regulatory executive function model in clinical psychosis. Schizophr. Res. 179, 75–84. 10.1016/j.schres.2016.09.032 27670237

[B62] Sellers R. Wells A. Morrison A. P. (2018). Are experiences of psychosis associated with unhelpful metacognitive coping strategies? A systematic review of the evidence. Clin. Psychol. Psychother. 25, 31–49. 10.1002/cpp.2132 28833863

[B63] Spada M. M. Mohiyeddini C. Wells A. (2008a). Measuring metacognitions associated with emotional distress: factor structure and predictive validity of the metacognitions questionnaire 30. Pers. Individ. Dif. 45, 238–242. 10.1016/j.paid.2008.04.005

[B64] Spada M. M. Nikčević A. V. Moneta G. B. Wells A. (2008b). Metacognition, perceived stress, and negative emotion. Pers. Individ. Dif. 44, 1172–1181. 10.1016/j.paid.2007.11.010

[B65] Strodl E. Stewart L. Mullens A. B. Deb S. (2015). Metacognitions mediate HIV stigma and depression/anxiety in men who have sex with men living with HIV. Health Psychol. Open. 2, 1–11. 10.1177/2055102915581562 28070355 PMC5193308

[B66] Sun X. Zhu C. So S. H. W. (2017). Dysfunctional metacognition across psychopathologies: a meta-analytic review. Eur. Psychiatry 45, 139–153. 10.1016/j.eurpsy.2017.05.029 28763680

[B67] Toffalini E. Veltri A. Cornoldi C. (2015). Metacognitive aspects influence subjective well-being in parents of children with cancer. Psychooncology 24, 175–180. 10.1002/pon.3622 25044029

[B68] Wells A. (2000a). Emotional Disorders and Metacognition: Innovative Cognitive Therapy. Chichester: John Wiley and Sons.

[B69] Wells A. (2000b). Metacognitive Therapy for Anxiety and Depression. New York, NY: The Guilford Press.

[B70] Wells A. Carter K. (2001). Further tests of a cognitive model of generalized anxiety disorder: metacognitions and worry in GAD, panic disorder, social phobia, depression, and nonpatients. Behav. Ther. 32, 85–102. 10.1016/S0005-7894(01)80045-9

[B71] Wells A. Cartwright-Hatton S. (2004). A short form of the metacognitions questionnaire: properties of the MCQ-30. Behav. Res. Ther. 42, 385–396. 10.1016/S0005-7967(03)00147-5 14998733

[B72] Wells A. Matthews G. (1996). Anxiety and cognition. Curr. Opin. Psychiatry 9, 422–426. 10.1097/00001504-199611000-00011

[B73] Wells A. Papageorgiou C. (1998). Relationships between worry, obsessive–compulsive symptoms and meta-cognitive beliefs. Behav. Res. Ther. 36, 899–913. 10.1016/S0005-7967(98)00070-9 9701864

[B74] Wells A. Purdon C. L. (1999). Metacognition and cognitive-behaviour therapy: a special issue. Clin. Psychol. Psychother. 6, 71–72. 10.1002/(SICI)1099-0879(199905)6:2<71::AID-CPP186>3.0.CO;2-G

[B75] World Health Organization (1992). The ICD-10 Classification of Mental And Behavioural Disorders: Clinical Descriptions and Diagnostic Guidelines. Geneva: World Health Organization.

[B76] Yilmaz A. E. Gençöz T. Wells A. (2011). The temporal precedence of metacognition in the development of anxiety and depression symptoms in the context of life-stress: a prospective study. J. Anxiety Dis. 25, 389–396. 10.1016/j.janxdis.2010.11.001 21144700

[B77] Ziadni M. S. Sturgeon J. A. Darnall B. D. (2018). The relationship between negative metacognitive thoughts, pain catastrophizing and adjustment to chronic pain. Eur. J. Pain. 22, 756–762. 10.1002/ejp.1160. 29214679 PMC5854507

